# Supplementary material for: Seipin forms a flexible cage at lipid droplet formation sites
Source: Nat Struct Mol Biol. 2022 Feb 24;29(3):194–202. doi: 10.1038/s41594-021-00718-y (PMC8930772; doi:10.1038/s41594-021-00718-y)
Supplement: Supplementary file 1 — Supplementary Tables 1 and 2. [file 41594_2021_718_MOESM1_ESM.pdf]

nature portfolio

https://doi.org/10.1038/s41594-021-00718-y

Supplementary information

Seipin forms a flexible cage at lipid droplet formation sites

In the format provided by the authors and unedited

Supplementary Information

Seipin forms a flexible cage at lipid droplet formation sites

Henning Arlt[1,2,3], Xuewu Sui[1,2], Brayden Folger[4], Carson Adams[5], Xiao Chen[4], Roman Remme[6], Fred A. Hamprecht[6], Frank DiMaio[5], Maofu Liao[2], Joel M. Goodman[4,*], Robert V. Farese, Jr.[1,2,7,*] & Tobias C. Walther[1,2,3,7,*]

[1]Department of Molecular Metabolism, Harvard T. H. Chan School of Public Health, Boston, MA, 02115 USA

[2]Department of Cell Biology, Harvard Medical School, Boston, MA, 02115 USA

[3]Howard Hughes Medical Institute, Boston, MA, 02115 USA

[4]Department of Pharmacology, University of Texas Southwestern Medical School, Dallas, TX 75390-9041, USA

[5]Department of Biochemistry and Institute of Protein Design, University of Washington, Seattle, WA, 98195 USA

[6]Heidelberg Collaborative for Image Processing, Interdisciplinary Center for Scientific Computing, Heidelberg University, 69120 Heidelberg, Germany

[7]Broad Institute of Harvard and MIT, Cambridge, MA, 02124 USA

[*] These authors contributed equally

Correspondence should be addressed to Joel M. Goodman (Joel.Goodman@UTSouthwestern.edu), Robert Farese, Jr. (robert@hsph.harvard.edu) and Tobias C. Walther (twalther@hsph.harvard.edu)

Table S1. Plasmids used in this study

Name	Description	Source
pSK^{-}-NAT-PGK		this study
pBMF1	*SEI1pr-SEI1-13xmyc::HIS3*	this study
pHA144	pET28a *SEI1(47-235) R178A-6xHis*	this study
pHA147	pET28a *SEI1(47-235)-6xHis*	this study
pHA234	pRS416 *ADHpr-GFP*	this study
pHA236	pRS416 *ADHpr-SEI1 (S33I Y37A Y41A)-GFP (Patch1)*	this study
pHA238	pRS416 *ADHpr-SEI1 (S33I Y37A Y41A M240G Y248I F255R I259K)-GFP (Patch1+2)*	this study
pHA240	pRS416 *ADHpr-SEI1(M240G Y248I F255R I259K)-EGFP (Patch2)*	this study
pHA246	pRS416 *ADHpr-SEI1-GFP*	this study
pHA247	pRS416 *ADHpr-SEI1 (R178A)-GFP*	this study
pHA249	pRS416 *ADHpr-SEI1(Q114A)-EGFP*	this study
pHA250	pRS416 *ADHpr-SEI1(E172A)-EGFP*	this study
pHA251	pRS416 *ADHpr-SEI1(E172A R178A)-EGFP*	this study
pHA252	pRS416 *ADHpr-SEI1(Q114A E172A R178A)-EGFP*	this study
pHA253	pRS416 *ADHpr-SEI1(Q114A E172A)-EGFP*	this study
pHA254	pRS416 *ADHpr-SEI1(Q114A R178A)-EGFP*	this study
pHA279	pRS416 *ADH1pr-dm seipin-EGFP*	this study
pHA406	pRS416 *ADHpr-SEI1 Δ169-173 -GFP*	this study
pHA407	pRS416 *ADHpr-SEI (169,172,173 to A) -GFP*	this study
pHA410	pRS416 *ADHpr-human seipin isoform 2 (1-300 aa) -GFP*	this study
pHA412	pRS416 *ADHpr-dm lumenal- yeast TM-GFP*	this study
pHA413	pRS416 *ADHpr-human seipin-yeast TM-GFP*	this study
pHA418	pRS416 *ADHpr-yeast lumenal-human TM-GFP*	this study
pHA432	pRS416 *ADHpr-SEI1 (167-185 7xA)-GFP*	this study
pHA433	pRS416 *ADHpr-SEI1 (Δ167-174)-GFP*	this study
pHA444	pRS416 *ADHpr-yeast lumenal-dm TM-GFP*	this study
pHA445	pRS416 *ADHpr-SEI1 (shuffled-switch)-GFP*	this study
pHA446	pRS416 *ADHpr-SEI1 (Δ-switch)-GFP*	this study
pHA523	pRS416 *ADHpr-SEI1 (C260L S266L T269I)-GFP*	this study
pHA525	pRS416 *ADHpr-SEI1 shuffled TMD N-GFP*	this study
pHA526	pRS416 *ADHpr-SEI1 shuffled TMD C-GFP*	this study
pHA527	pRS416 *ADHpr-SEI1 shuffled TMD N+C-GFP*	this study
pHA528	pRS416 *ADHpr-SEI1 TMD N FIT2-GFP*	this study
pHA529	pRS416 *ADHpr-SEI1 TMD C FIT2-GFP*	this study
pHA530	pRS416 *ADHpr-SEI1 TMD N+C FIT2-GFP*	this study
pTW185	ssHDEL-MARS*::LEU2*	

Italic font indicates gene or promoter information.

Table S2. Yeast *S. cerevisiae* strains used in this study

Name	Genotype	Source
BY4741	*MATa his3Δ1 leu2Δ0 met15Δ0 ura3Δ0*	Euroscarf
HAY60	BY4741; *kanMX::GALpr)-FLD1-3xFLAG-TEV-ProtA::hygR natNT2::GAL1pr-LDB16*	this study
TWY2143	BY4741; *sei1::kanMX*	Euroscarf
W303-1A	*MATa leu2-3,112 trp1-1 can1-100 ura3-1 ade2-1 his3-11,15*	[35]
W303-1A *sei1Δ*	W303-1A; *sei1::hygR*	[47]
W303-1A *sei1Δ pln1Δ*	W303-1A; *sei1::hygR pln1::HIS3MX6*	[47]
W303-1A *pln1Δ*	W303-1A; *pln1::hygR*	[47]
SEI1-myc	W303-1A *pln1Δ; SEI1::13xmyc-HIS3*	this study
ldb16Δ SEI1-myc	W303-1A *pln1Δ; ldb16:kanMX:natNT1::PGK1pr- SEI1::13xmyc-HIS3*	this study
R178A	W303-1A *pln1Δ; SEI1 (R178A)::13xmyc-HIS3*	this study
Q114A	W303-1A *pln1Δ; SEI1 (Q114A)::13xmyc-HIS3*	this study
E172A	W303-1A *pln1Δ; SEI1 (E172A)::13xmyc-HIS3*	this study
Q114A R178A	W303-1A *pln1Δ; SEI1 (Q114A R178A)::13xmyc-HIS3*	this study
E172A R178A	W303-1A *pln1Δ; SEI1 (E172A R178A)::13xmyc-HIS3*	this study
Q114A E172A	W303-1A *pln1Δ; SEI1 (Q114A E172A)::13xmyc-HIS3*	this study
Q114A E172A R178A	W303-1A *pln1Δ; SEI1 (Q114A E172A R178A)::13xmyc-HIS3*	this study
PGK1pr-R178A	W303-1A *pln1Δ; natNT1::PGK1pr-SEI1 (R178A)::13xmyc-HIS3*	this study
R178A PGK1pr-LDB16	W303-1A *pln1Δ; natNT1::PGK1pr-SEI1 (R178A)::13xmyc-HIS3*	this study
Patch1	W303-1A *pln1Δ; PGK1pr-SEI1 (Patch1)-13xmyc::HIS3*	this study
Patch1 R178A	W303-1A *pln1Δ; PGK1pr-SEI1 (Patch1 R178A)-13xmyc::HIS3*	this study
Patch2	W303-1A *pln1Δ; hygR PGK1pr-SEI1 (Patch2)-13xmyc::HIS3*	this study
Patch2 R178A	W303-1A *pln1Δ; PGK1pr-SEI1 (Patch2 R178A)-13xmyc::HIS3*	this study
Patch1+2	W303-1A *pln1Δ; PGK1pr-SEI1 (Patch1+2)-13xmyc::HIS3*	this study
Patch1+2 R178A	W303-1A *pln1Δ; PGK1pr-SEI1 (Patch1+2 R178A)-13xmyc::HIS3*	this study
Sei1 (TM-N-FIT2)	W303-1A *pln1Δ; PGK1pr-SEI1 (TMD-N)-13xmyc::HIS3*	this study
Sei1 (TM-C-FIT2)	W303-1A *pln1Δ; PGK1pr-SEI1 (TMD-C)-13xmyc::HIS3*	this study
Sei1 (TM-NC-FIT2)	W303-1A *pln1Δ, PGK1pr-SEI1 (TMD-N+C)-13xmyc::HIS3*	this study
Sei1 (shuffled TM-N)	W303-1A *pln1Δ; PGK1pr-SEI1 (shTMD-N)-13xmyc::HIS3*	this study
Sei1 (shuffled TM-C)	W303-1A *pln1Δ; PGK1pr-SEI1 (shTMD-C)-13xmyc::HIS3*	this study
Sei1 (shuffled TM-NC)	W303-1A *pln1Δ; PGK1pr-SEI1 (shTMD-N+C)-13xmyc::HIS3*	this study
C260L S266L T269I	W303-1A *pln1Δ; PGK1pr-SEI1 (C260L S266L T269I)-13xmyc::HIS3*	this study
Δ-switch	W303-1A *pln1Δ; hygR natNT1::PGK1pr-SEI1 (Δ-switch)-*	this study
shuffled-switch	W303-1A *pln1Δ; natNT1::PGK1pr-SEI1 (shuffled-switch)-13xmyc ::HIS3*	this study
169,172,173 to A	W303-1A *pln1Δ; natNT1::PGK1pr-SEI1 (3xA)::13xmyc-HIS3*	this study
167-185 7xA	W303-1A *pln1Δ; natNT1::PGK1pr-SEI1 (7xA)::13xmyc-HIS3*	this study
Δ169-173	W303-1A *pln1Δ; natNT1::PGK1pr-SEI1 (Δ169-173)::13xmyc-HIS3*	this study
Δ167-174	W303-1A *pln1Δ; natNT1::PGK1pr-SEI1 (Δ167-174)::13xmyc-HIS3*	this study

Italic font indicates gene or promoter information.
