# Supplementary material for: Seipin forms a flexible cage at lipid droplet formation sites
Source: Nat Struct Mol Biol. 2022 Feb 24;29(3):194–202. doi: 10.1038/s41594-021-00718-y (PMC8930772; doi:10.1038/s41594-021-00718-y)
Supplement: Supplementary file 2 — Reporting Summary [file 41594_2021_718_MOESM2_ESM.pdf]

nature portfolio

Corresponding author(s): Dr. Tobias C. Walther; Dr. Robert V. Farese, Jr.; Dr. Joel M. Goodman

Last updated by author(s): Aug 10, 2021

Reporting Summary

Nature Portfolio wishes to improve the reproducibility of the work that we publish. This form provides structure for consistency and transparency in reporting. For further information on Nature Portfolio policies, see our Editorial Policies and the Editorial Policy Checklist.

Statistics

For all statistical analyses, confirm that the following items are present in the figure legend, table legend, main text, or Methods section.

n/a	Confirmed	
☐	☒	The exact sample size (*n*) for each experimental group/condition, given as a discrete number and unit of measurement
☐	☒	A statement on whether measurements were taken from distinct samples or whether the same sample was measured repeatedly
☐	☒	The statistical test(s) used AND whether they are one- or two-sided<br>*Only common tests should be described solely by name; describe more complex techniques in the Methods section.*
☒	☐	A description of all covariates tested
☒	☐	A description of any assumptions or corrections, such as tests of normality and adjustment for multiple comparisons
☐	☒	A full description of the statistical parameters including central tendency (e.g. means) or other basic estimates (e.g. regression coefficient) AND variation (e.g. standard deviation) or associated estimates of uncertainty (e.g. confidence intervals)
☐	☒	For null hypothesis testing, the test statistic (e.g. *F*, *t*, *r*) with confidence intervals, effect sizes, degrees of freedom and *P* value noted<br>*Give P values as exact values whenever suitable.*
☒	☐	For Bayesian analysis, information on the choice of priors and Markov chain Monte Carlo settings
☒	☐	For hierarchical and complex designs, identification of the appropriate level for tests and full reporting of outcomes
☒	☐	Estimates of effect sizes (e.g. Cohen's *d*, Pearson's *r*), indicating how they were calculated

Our web collection on statistics for biologists contains articles on many of the points above.

Software and code

Policy information about availability of computer code

Data collection	SerialEM version 3.6; NIS-Elements AR (v4.51.01)
Data analysis	Phenix (v 1.18); Coot (v 0.95); MotionCor2 (v 1.1.0); CTFFIND4 (v 4.1.5); SAMUEL (v 17.05); SamViewer (v 16.01); SPIDER (v 17.05); RELION 3.0; Resmap (v 1.1.4); bfactor (v 1.03); Chimera (v 1.13); ImageJ (v 2.0); GraphPad PRISM (v 9); Image Studio (v 5.2.5); custom code to segment cells is made publicly available at: https://github.com/hci-unihd/YeastCellSeg

For manuscripts utilizing custom algorithms or software that are central to the research but not yet described in published literature, software must be made available to editors and reviewers. We strongly encourage code deposition in a community repository (e.g. GitHub). See the Nature Portfolio guidelines for submitting code & software for further information.

Data

Policy information about availability of data

All manuscripts must include a data availability statement. This statement should provide the following information, where applicable:
- Accession codes, unique identifiers, or web links for publicly available datasets
- A description of any restrictions on data availability
- For clinical datasets or third party data, please ensure that the statement adheres to our policy

EM density map and protein coordinates were deposited to Electron Microscopy Data bank (ID EMD-24674) and Protein Data Bank (PDB ID-7RSL), respectively.

Field-specific reporting

Please select the one below that is the best fit for your research. If you are not sure, read the appropriate sections before making your selection.

☒ Life sciences ☐ Behavioural & social sciences ☐ Ecological, evolutionary & environmental sciences

For a reference copy of the document with all sections, see nature.com/documents/nr-reporting-summary-flat.pdf

Life sciences study design

All studies must disclose on these points even when the disclosure is negative.

Sample size	No statistical methods were used to predetermine sample size. Sample sizes were chosen based on similar studies in the field.
Data exclusions	No data were excluded from analyses.
Replication	Each experiment was repeated at least two times in independent experiments. All attempts at replication were successful.
Randomization	Not applicable to our study, as there was no assignment to different groups.
Blinding	Not applicable to our study, as there was no assignment into groups.

Reporting for specific materials, systems and methods

We require information from authors about some types of materials, experimental systems and methods used in many studies. Here, indicate whether each material, system or method listed is relevant to your study. If you are not sure if a list item applies to your research, read the appropriate section before selecting a response.

Materials & experimental systems

n/a	Involved in the study	
☐	☒	Antibodies
☒	☐	Eukaryotic cell lines
☒	☐	Palaeontology and archaeology
☒	☐	Animals and other organisms
☒	☐	Human research participants
☒	☐	Clinical data
☒	☐	Dual use research of concern

Methods

n/a	Involved in the study	
☒	☐	ChIP-seq
☒	☐	Flow cytometry
☒	☐	MRI-based neuroimaging

Antibodies

Antibodies used	anti-myc monoclonal 9E10 (Cat# MA1-980; Thermofisher); anti-G6PDH (Cat#A-9521; Sigma); goat anti-mouse-IRDye (Cat# 926-32210; Licor); goat anti-rabbit-IRDye (Cat# 926-32211, Licor); m-IgGκ BP-HRP (Cat# sc-516102; Santa Cruz Biotechnology); mouse anti-rabbit IgG-HRP (Cat# sc-2357; Santa Cruz Biotechnology); Mouse monoclonal anti-Pgk1 (Cat# 459250; Invitrogen); Mouse monoclonal anti-FLAG (Cat# F1804; Sigma); anti-Ldb16 rabbit polyclonal (Wang et al., 2014), gift from Chao-Wen Wang (Institute of Plant and Microbial Biology, Academia Sinica, Taipei City).
Validation	anti-Ldb16 antibodies were verified on western blots of cell lysate from WT vs. ldb16Δ cells and were previously validated in Wang et al., 2014. Validations of commercially available antibodies can be found at the manufacturers sites: anti-myc monoclonal (https://www.thermofisher.com/antibody/product/c-Myc-Antibody-clone-9E10-Monoclonal/MA1-980); anti-G6PDH (https://www.sigmaaldrich.com/US/en/product/sigma/a9521); anti-Pgk1 (https://www.thermofisher.com/antibody/product/PGK1-Antibody-clone-22C5D8-Monoclonal/459250); anti-FLAG (https://www.sigmaaldrich.com/US/en/product/sigma/f3165).

March 2021
