# Supplementary figures and images for: Seipin forms a flexible cage at lipid droplet formation sites
Source: Nat Struct Mol Biol. 2022 Feb 24;29(3):194–202. doi: 10.1038/s41594-021-00718-y (PMC8930772; doi:10.1038/s41594-021-00718-y)

## Source Data Figure 2e

high resolution

low resolution merge  
with marker

WT

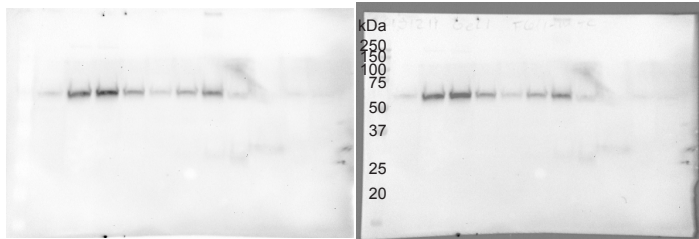

R178A

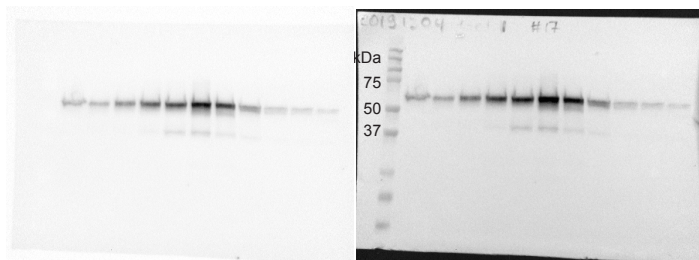

Supplement: Source Data Fig. 2 — Unprocessed western blots. [file 41594_2021_718_MOESM5_ESM.pdf]

Source Data Figure 4e

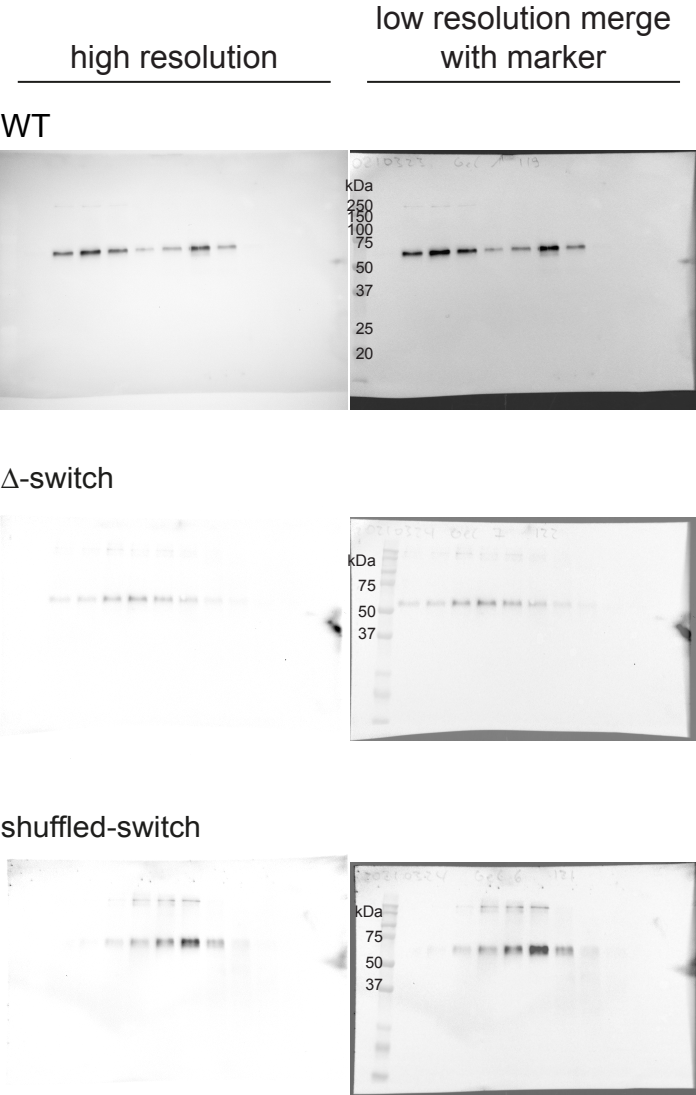

Supplement: Source Data Fig. 4 — Unprocessed western blots. [file 41594_2021_718_MOESM9_ESM.pdf]

Source Data Figure 9b

aMyc

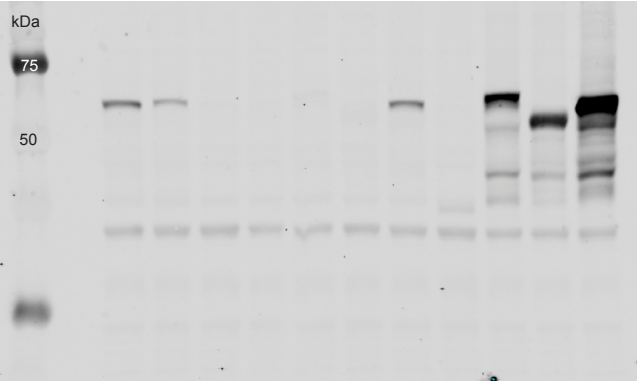

aG6PDH

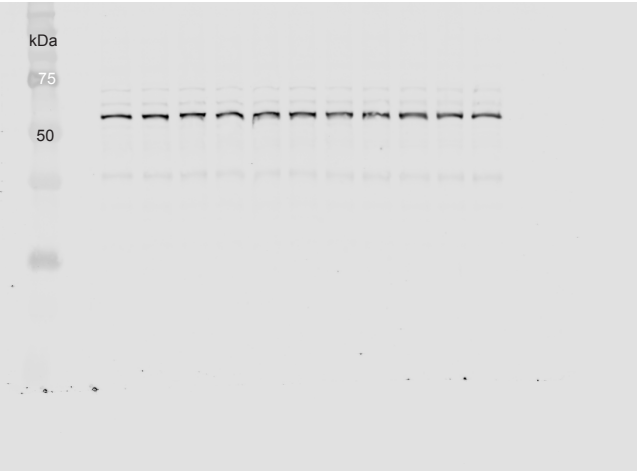

Supplement: Source Data Extended Data Fig. 9 — Unprocessed western blots. [file 41594_2021_718_MOESM20_ESM.pdf]
