# Supplementary material for: Seipin forms a flexible cage at lipid droplet formation sites
Source: Nat Struct Mol Biol. 2022 Feb 24;29(3):194–202. doi: 10.1038/s41594-021-00718-y (PMC8930772; doi:10.1038/s41594-021-00718-y)

Source Data Extended Figure 3f

| high resolution | low resolution merge with marker |
|---|---|

WT

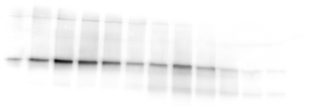

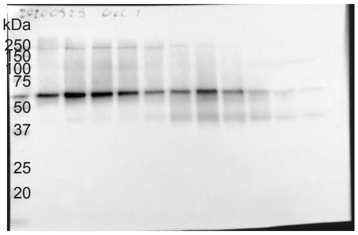


R178A

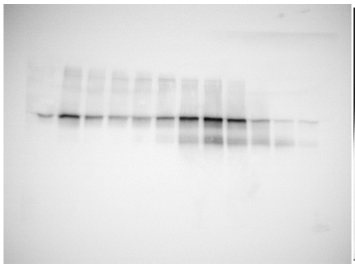

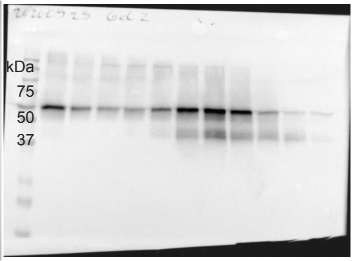


Patch 1

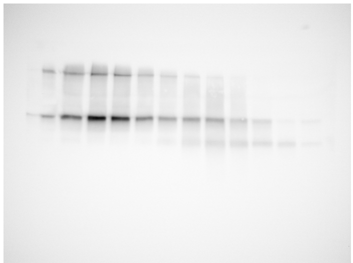

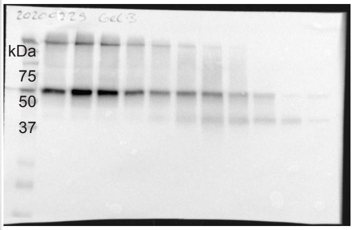


Patch 2

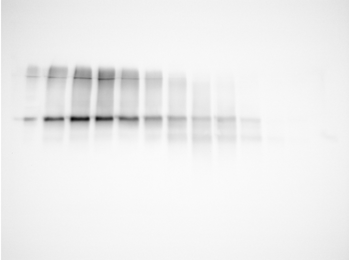

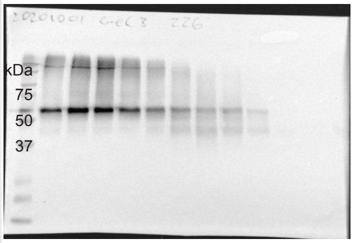


Patch 1+2

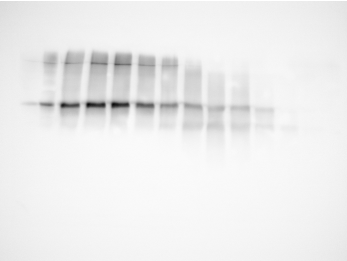

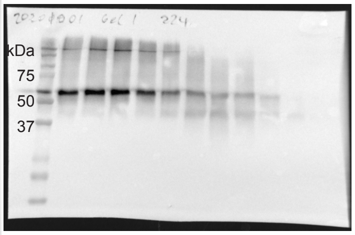

Supplement: Source Data Fig. 3 — Unprocessed western blots. [file 41594_2021_718_MOESM7_ESM.pdf]
