# Supplementary material for: Seipin forms a flexible cage at lipid droplet formation sites
Source: Nat Struct Mol Biol. 2022 Feb 24;29(3):194–202. doi: 10.1038/s41594-021-00718-y (PMC8930772; doi:10.1038/s41594-021-00718-y)

Source Data Extended Figure 1d

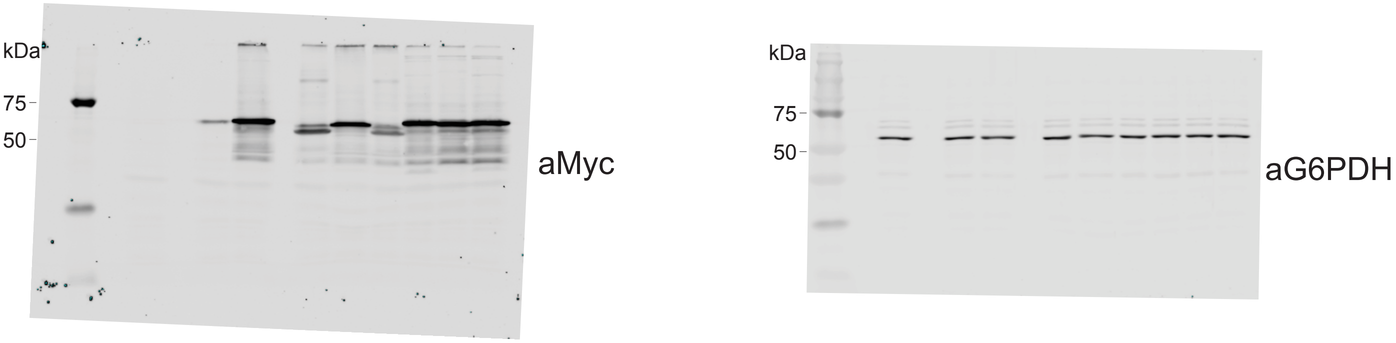

Source Data Extended Figure 1e

high resolution	low resolution merge with marker	high resolution	low resolution merge with marker

WT

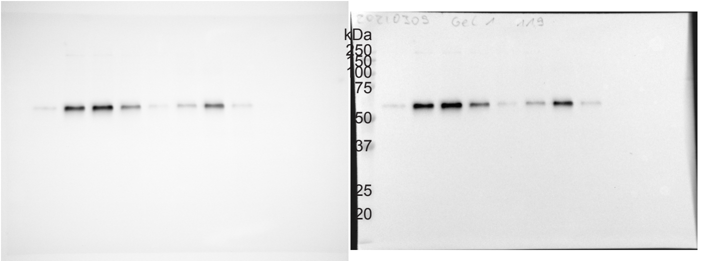

TM-N-FIT2

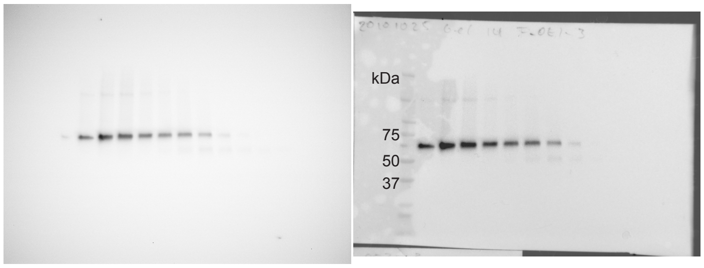

TM-C-FIT2

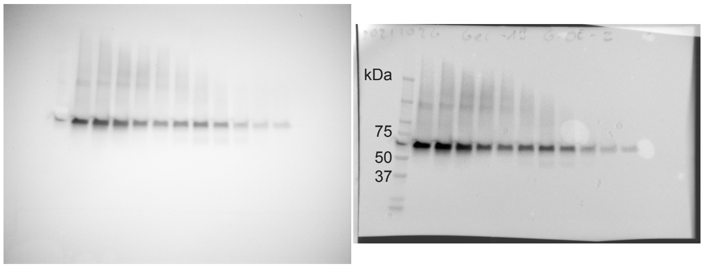

TM-NC-FIT2

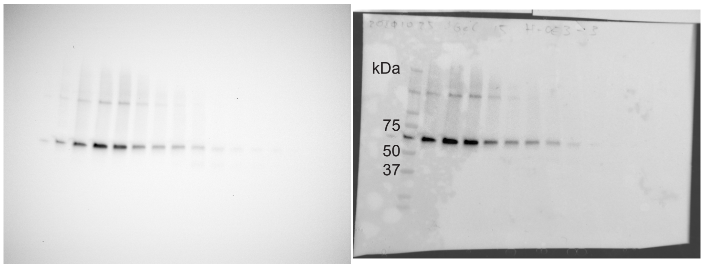

shuffled TM-N

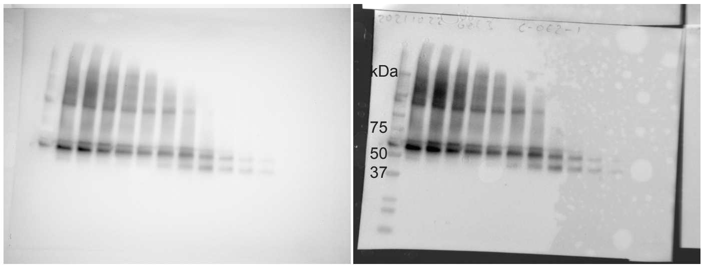

shuffled TM-C

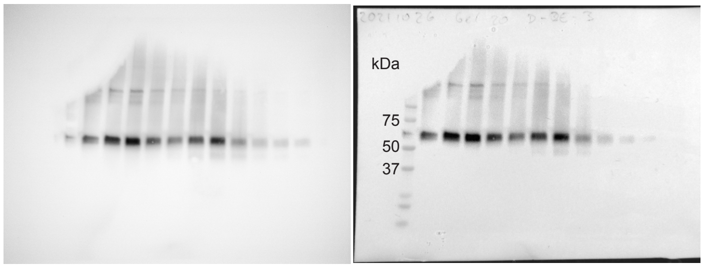

shuffled TM-NC

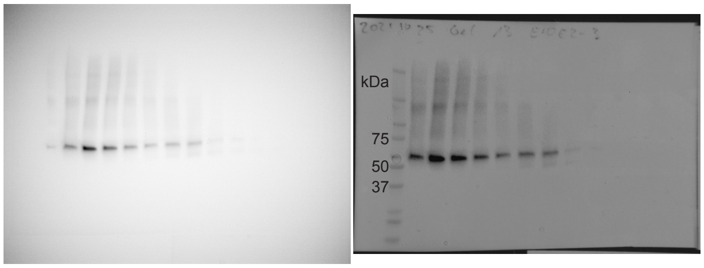

Supplement: Source Data Extended Data Fig. 1 — Unprocessed western blots. [file 41594_2021_718_MOESM11_ESM.pdf]
