# Supplementary material for: Seipin forms a flexible cage at lipid droplet formation sites
Source: Nat Struct Mol Biol. 2022 Feb 24;29(3):194–202. doi: 10.1038/s41594-021-00718-y (PMC8930772; doi:10.1038/s41594-021-00718-y)

Source Data Extended Figure 2b

Coomassie

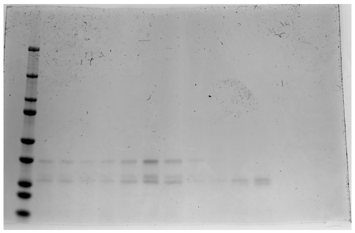

high resolution | low resolution merge with marker

aFlag

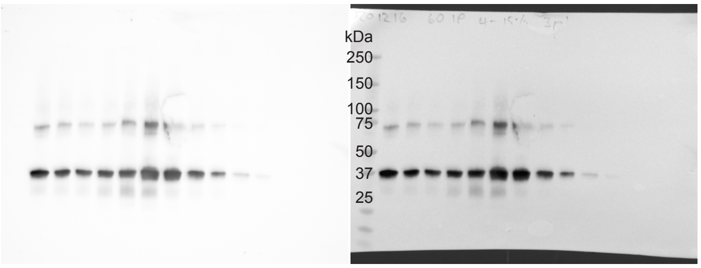

aLdb16

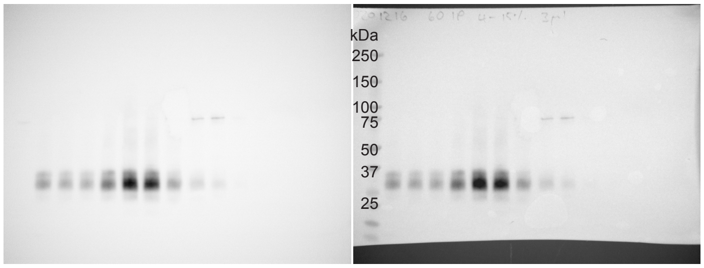

Supplement: Source Data Extended Data Fig. 2 — Unprocessed western blots. [file 41594_2021_718_MOESM12_ESM.pdf]
