# Supplementary material for: Seipin forms a flexible cage at lipid droplet formation sites
Source: Nat Struct Mol Biol. 2022 Feb 24;29(3):194–202. doi: 10.1038/s41594-021-00718-y (PMC8930772; doi:10.1038/s41594-021-00718-y)

## Source Data Extended Figure 4a

### aMyc

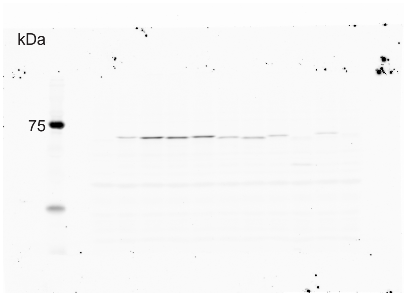


### aG6PDH

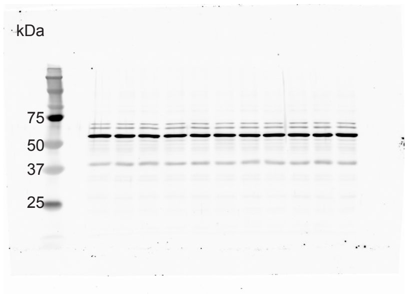


## Source Data Extended Figure 4e

high resolution | low resolution merge with marker

### aGFP

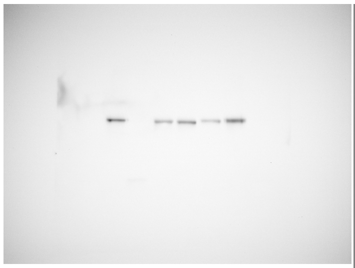

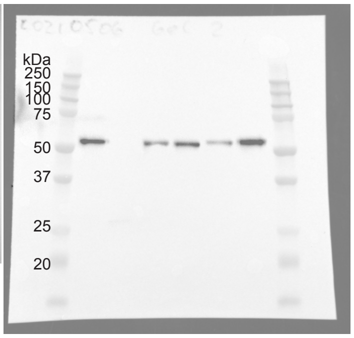


### aLdb16

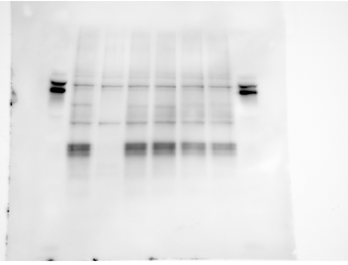

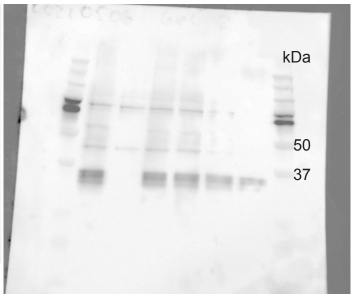


### aPgk1

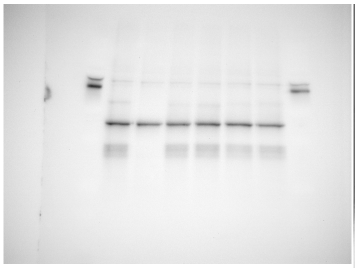

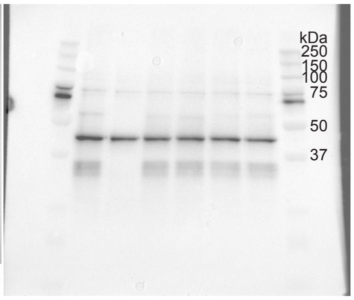

Supplement: Source Data Extended Data Fig. 4 — Unprocessed western blots. [file 41594_2021_718_MOESM14_ESM.pdf]
