# Supplementary material for: Seipin forms a flexible cage at lipid droplet formation sites
Source: Nat Struct Mol Biol. 2022 Feb 24;29(3):194–202. doi: 10.1038/s41594-021-00718-y (PMC8930772; doi:10.1038/s41594-021-00718-y)

Source Data Extended Figure 5a

aMyc

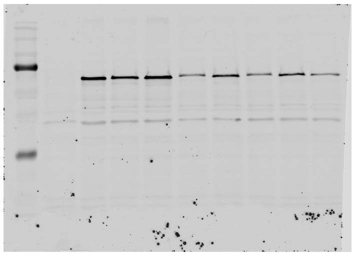

aG6PDH

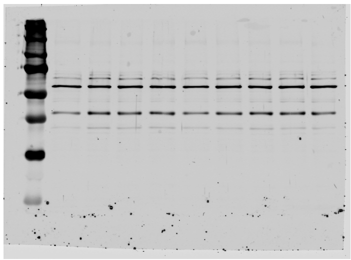

Source Data Extended Figure 5b

high resolution	low resolution merge with marker

WT

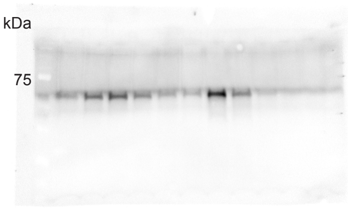

ldb16Δ

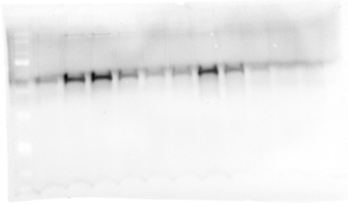

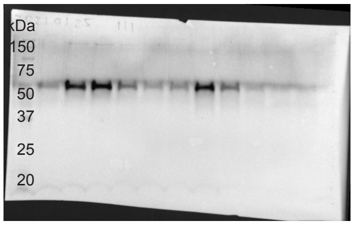

R178A PGK1pr-LDB16

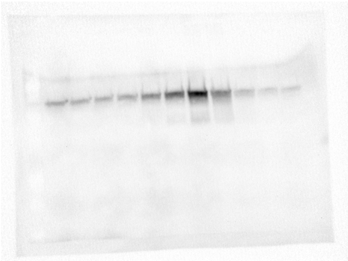

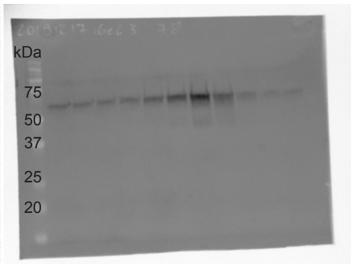

PGK1pr-R178

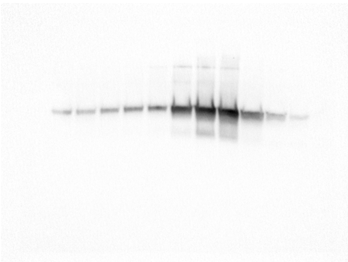

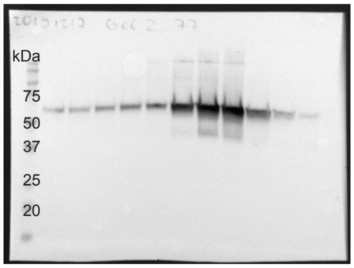

Source Data Extended Figure 5h

WT$_{(47\text{-}235)}$

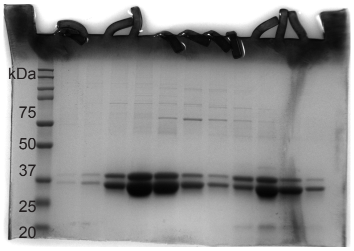

R178A$_{(47\text{-}235)}$

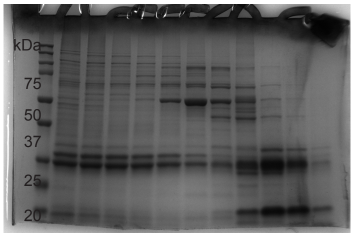

Supplement: Source Data Extended Data Fig. 5 — Unprocessed western blots. [file 41594_2021_718_MOESM16_ESM.pdf]
