# Supplementary material for: Seipin forms a flexible cage at lipid droplet formation sites
Source: Nat Struct Mol Biol. 2022 Feb 24;29(3):194–202. doi: 10.1038/s41594-021-00718-y (PMC8930772; doi:10.1038/s41594-021-00718-y)

Source Data Figure 6a

anti-G6PDH

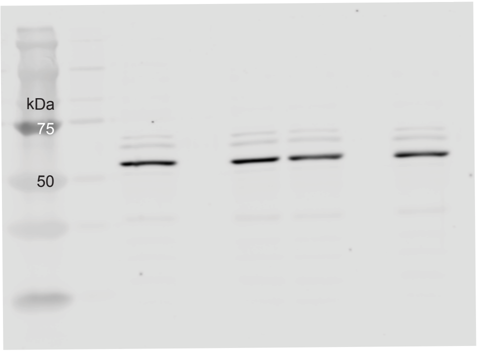

aMyc

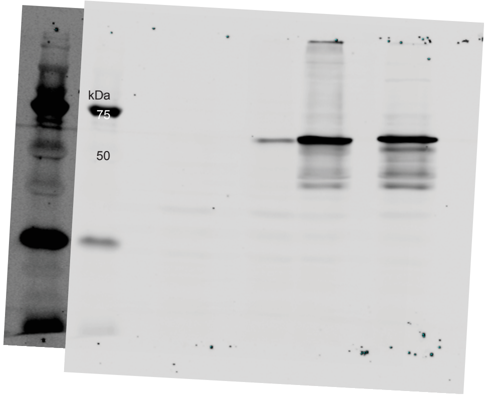

Source Data Figure 6b

high resolution

low resolution merge with marker

aFlag

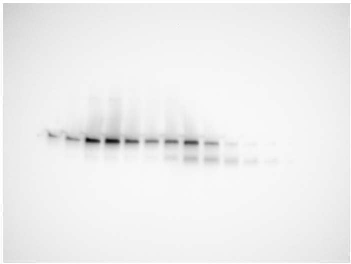

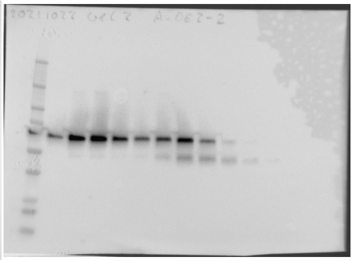

Supplement: Source Data Extended Data Fig. 6 — Unprocessed western blots. [file 41594_2021_718_MOESM18_ESM.pdf]
