# Supplementary material for: Seipin forms a flexible cage at lipid droplet formation sites
Source: Nat Struct Mol Biol. 2022 Feb 24;29(3):194–202. doi: 10.1038/s41594-021-00718-y (PMC8930772; doi:10.1038/s41594-021-00718-y)

Source Data Extended Figure 7a

aMyc

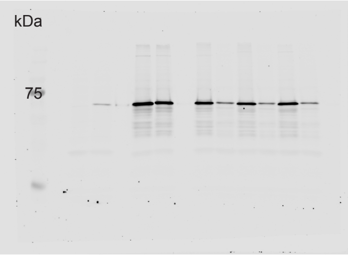

aG6PDH

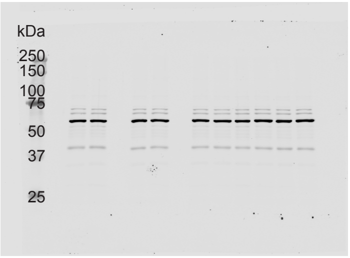

Source Data Extended Figure 7b

high resolution	low resolution merge with marker

Patch1 R178A

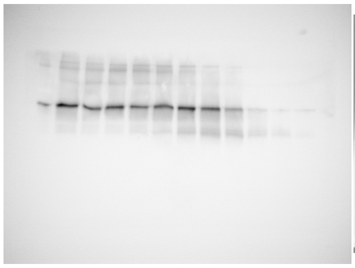

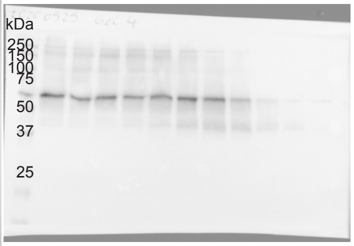

Patch2 R178A

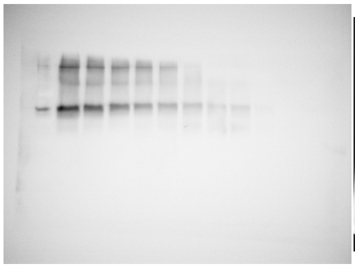

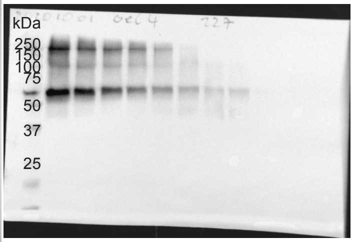

Patch1+2 R178A

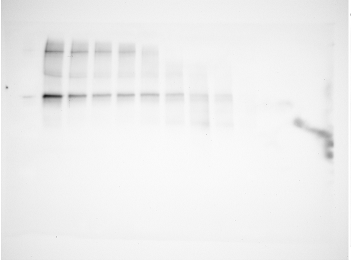

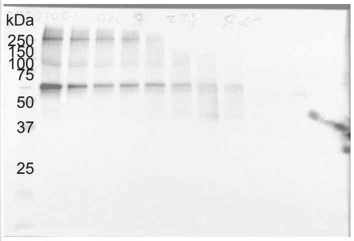

Source Data Extended Figure 7d

high resolution	low resolution merge with marker

aPgk1

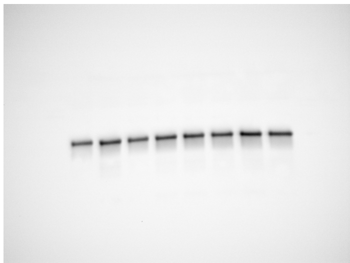

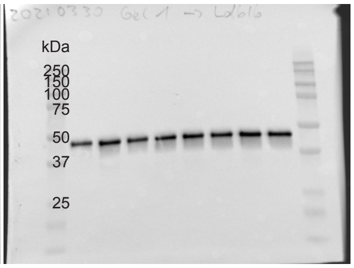

aLdb16

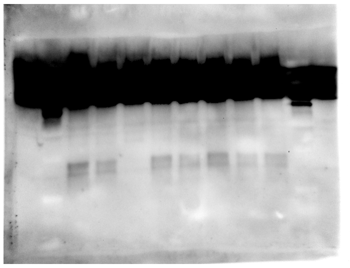

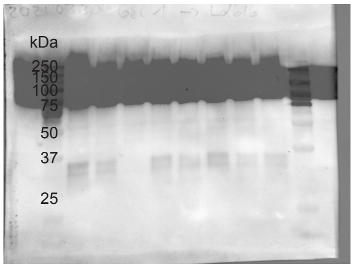

aMyc

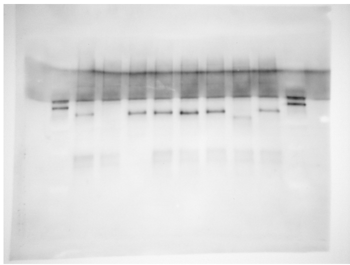

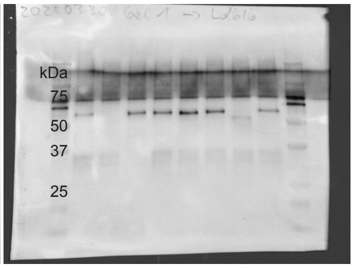

Source Data Extended Figure 7e

Load-aMyc

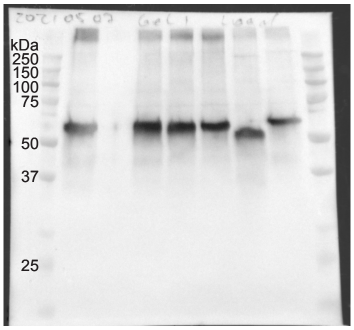

Load-aLdb16

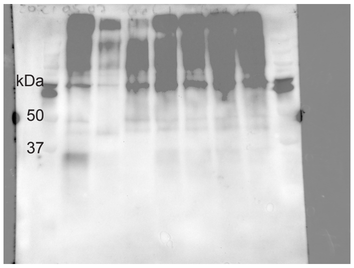

IP-aMyc

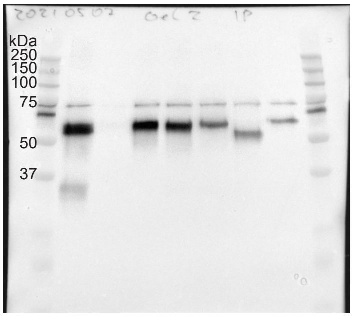

IP-aLdb16

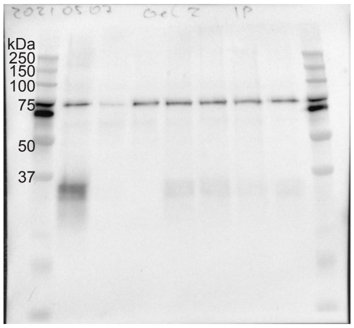

Supplement: Source Data Extended Data Fig. 7 — Unprocessed western blots. [file 41594_2021_718_MOESM19_ESM.pdf]
